# Association of physical activity with chronic kidney disease: a systematic review and dose-response meta-analysis

**DOI:** 10.18632/aging.103747

**Published:** 2020-10-07

**Authors:** Yongjian Zhu, Yongjun Bu, Guofu Zhang, Shibin Ding, Desheng Zhai, Zhongxiao Wan, Zengli Yu

**Affiliations:** 1 School of Public Health, Xinxiang Medical University, Xinxiang 453003, China; 2 School of Public Health, Zhengzhou University, Zhengzhou 450001, China

**Keywords:** physical activity, chronic kidney disease, dose-response, systematic review, meta-analysis

## Abstract

Background: The relationship between physical activity (PA) and chronic kidney disease (CKD) risk was inconsistent. We therefore conducted a systematic review and dose-response meta-analysis to comprehensively evaluate the association of PA and CKD.

Results: A total of 14 studies from 13 articles with 353,975 participants were included. By comparing the highest vs. the lowest level of PA, we found that PA was inversely associated with CKD risk (odds ratio [OR] = 0.94, 95% confidence interval [CI] = 0.91–0.98). Seven studies from 6 articles were included in dose–response analysis. Restricted cubic splines showed no evidence of a nonlinear dose–response relationship of PA and CKD risk (*P*_nonlinearity_ = 0.135). The risk of CKD was reduced by 2% (OR = 0.98, 95% CI = 0.96–1.00) with each 10 metabolic equivalent h/week increment of PA.

Conclusions: The findings demonstrated that the higher level of PA might have a protective effect against the risk of CKD.

Methods: Electronic databases PubMed and Embase were searched up to March 11, 2020. Observational studies investigated the relationship between PA and CKD risk with estimated effects (relative risk, hazard ratio, or OR) with 95 % CI among adults were included.

## INTRODUCTION

Chronic kidney disease (CKD) which is characterized as the loss of renal structure and function [1] has been recognized as one of the major public health. The global burden disease study 2017 found that CKD ranked as the 15^th^ leading cause of death and resulted in more than 2.6 million deaths worldwide in 2017 [2]. Patients with CKD are at high risk of poor quality of life, anemia, infection, cognitive decline, bone disorders and fractures, as well as all-cause and cardiovascular mortality, which may result in huge social and economic burdens [3–7].

It is now identified that lack of physical activity (PA) is the fourth leading risk factor for global mortality [8]. The world health organization (WHO) suggested that PA is considered as a key contributor to prevent non-communicable diseases (NCDs) [9]. Previous evidence has indicated that a higher PA level was associated with reduced risk of cardiovascular disease, type 2 diabetes, and metabolic syndrome [10–13]. The WHO also recommend that adults aged 18-64 year old should do at least 150 minutes of moderate-intensity PA per week to reduce the risk of NCDs [14]. PA also has been hypothesized as a modifiable risk factor for the development of CKD. Over the past 2 decades, a number of epidemiological studies have assessed the potential relationship between PA and CKD risk in various study populations [15–27]. However, these results are still controversial. Therefore, we conducted the current systematic review and dose-response meta-analysis to comprehensively investigate the association of PA and CKD risk.

## RESULTS

### Characteristics of studies

**Figure 1:**
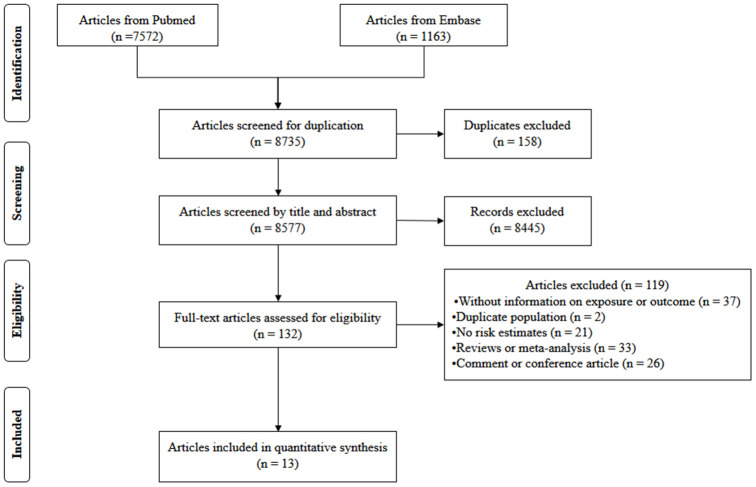
**Flow diagram of screened, excluded, and analyzed publications.**

The flow of study selection was presented in Figure 1. A total of 7,572 articles from PubMed and 1,163 articles from Embase were retrieved. After removing 158 duplicate publications, 8,577 articles were reviewed. Among them, 8,445 articles were excluded after the title and abstract review, leaving 132 articles for full-text review. Among them, 119 publications were excluded due to: reviews, no results provided the exposure or outcome, duplicate studies, no risk estimates, reviews or meta-analysis, and comment or conference article. Among the remaining 13 articles, one reported effect estimates for males and females, respectively [16]. Thus, totally 14 studies from 13 articles [15–27] including 353,975 participants were included.

The main characters of the included studies are presented in Supplementary Table 1. Among them, 6 were cohort studies, and 8 studies were cross-sectional studies. For the measurement of PA, eight studies used standardized questionnaire and others adopted self-reported results. For the definition of CKD, the 7 studies defined eGFR < 60 mL/min/1.73 m^2^ or less as CKD, and the other 7 studies defined decreased eGFR and/or microproteinuria as CKD.

### Quality assessment

In the six articles with a cohort design, the quality assessment was conducted based on Newcastle-Ottawa scale and suggested that two article with a score of 6, 1 article with 7, and 3 articles with 8 (Supplementary Table 2). In the seven articles with a cross-sectional design, all studies met at least six criteria according to the JBI checklists (Supplementary Table 3).

### High versus low PA analysis

**Figure 2:**
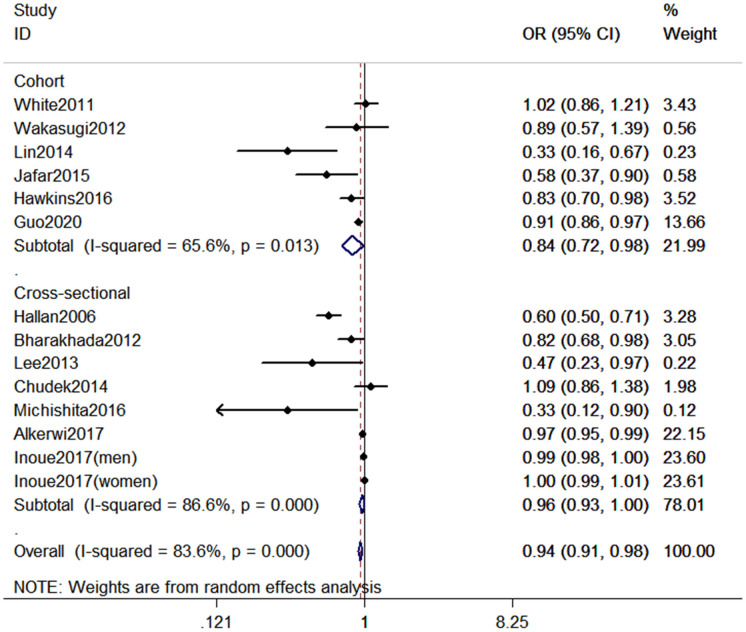
**Forest plot between highest vs. lowest level of PA and CKD risk.**

As shown in Figure 2, 14 studies (13 publications) [15–27] with 353,975 participants were included in the analysis of highest versus lowest PA in the odds of CKD. Compared to the lowest PA level, the highest PA level was associated with a decreased odds of CKD (Pooled odds ratio [OR] = 0.94, 95% confidence interval [CI] = 0.91–0.98, *P* < 0.001). The pooled estimates in cohort studies and cross-sectional studies were 0.84 (95% CI: 0.72-0.98, *P* = 0.013) and 0.96 (95% CI: 0.93-1.00, *P* < 0.001), respectively.

### Dose–response association between PA and the risk of CKD

**Figure 3:**
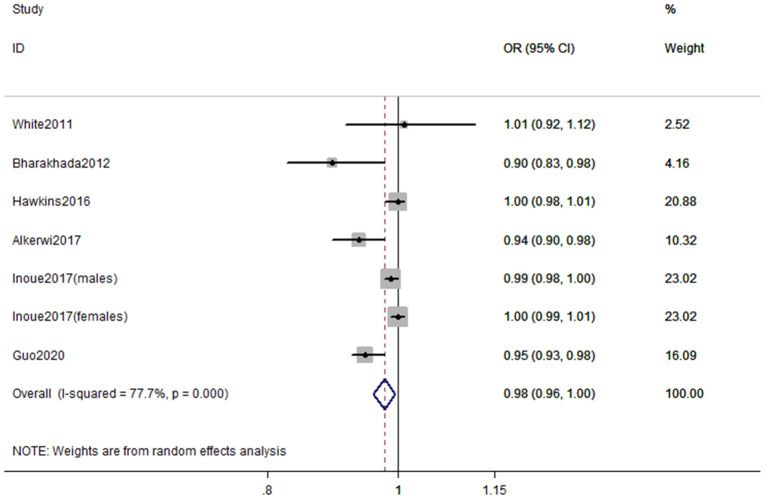
**Forest plot of study-specific risk estimates for CKD per 10 MET h/wk increment of PA.**

**Figure 4:**
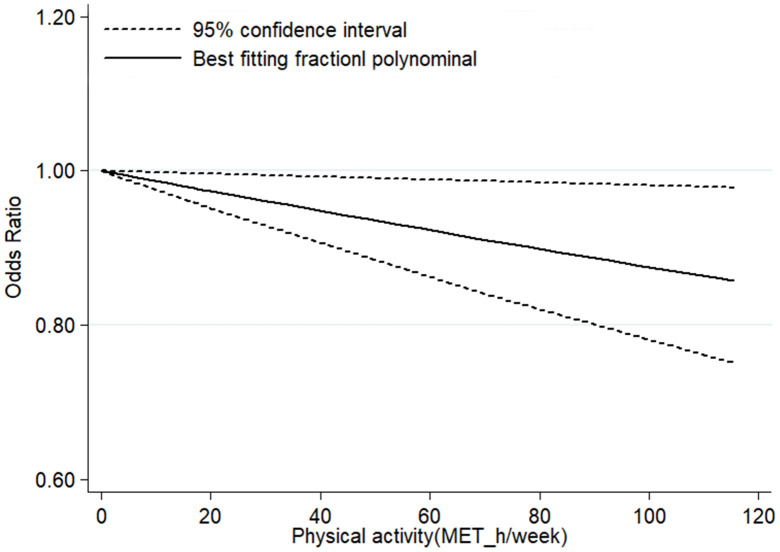
**Linear dose–response association between PA and risk of CKD.**

According to the inclusion criteria for dose-response analysis, 6 articles with 7 studies involving 218,228 participants [16, 17, 23, 24, 26, 27] were included in dose–response analyses. The pooled OR for the risk of CKD was 0.98 (95% CI, 0.96–1.00, *P* < 0.001) with each increment of 10 metabolic equivalent (MET) h/wk of PA (Figure 3). In these 7 studies, three studies (2 articles) [16, 24] only reported continuous risk estimates, which did not meet the requirements of nonlinear dose-response analysis. As a result, four studies were finally included in the nonlinear dose-response analysis. No evidence suggested that there is a nonlinear association between PA and CKD (*P*_nonlinearity_ = 0.135). We therefore used restricted cubic splines to model the linear dose-response relationship. A marginal negative linear relationship between PA and risk of CKD was observed (Figure 4).

### Meta-regression, subgroup, sensitivity analyses, and publication bias

**Figure 5:**
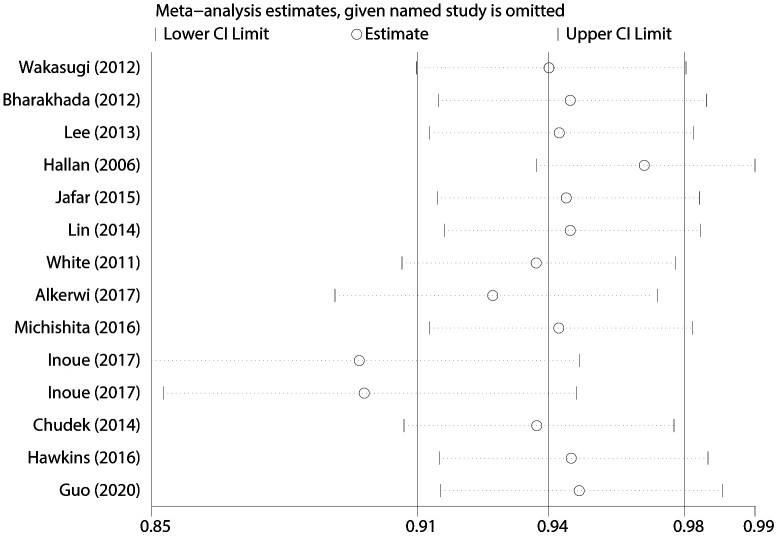
**“Leave-one-out” sensitivity analysis about PA and CKD risk.**

Meta-regression and subgroup analyses with geographic locations, sample size, study design, types of PA, and covariates (smoking, alcohol drinking, and body mass index [BMI]) showed non-significant impact on between-study heterogeneity (all *P* > 0.05) (Table 1). We also conducted “leave-one-out” sensitivity analyses and found that no individual study significantly affected the pooled OR, which indicated that our results were statistically robust (Figure 5). When excluded the study of Inoue et al. in which the PA intensity was assumed as 4.5 METs, the pooled estimate (OR: 0.94, 95% CI: 0.91-0.97) was also consistent with the main result.

**Table 1:** Subgroup analysis of highest vs. lowest PA and risk of CKD.

**Subgroups**	**No. of Participants**	**No. of studies**	**OR (95% CI)**	**Heterogeneity test**	
				***I*^2^ (%)**	***P***
All studies	353,975	14	0.94 (0.91-0.98)	83.6	< 0.001
**Geographic locations**					
Europe	76,005	4	0.85 (0.67-1.07)	90.8	< 0.001
America	2,435	1	0.83 (0.70-0.98)	N/A	N/A
Asia	264,569	8	0.97 (0.91-1.00)	79.4	< 0.001
Australia	10,966	1	1.02 (0.86-1.21)	N/A	N/A
**Sample size**					
≥ 2000	350,373	10	0.94 (0.91-0.98)	84.2	< 0.001
< 2000	3,602	4	0.51 (0.26-1.01)	82.6	< 0.001
**Study design**					
Cohort	268,488	6	0.84 (0.72-0.98)	65.6	0.013
Cross-sectional	85,487	8	0.96 (0.93-1.00)	86.6	< 0.001
**Type of PA**					
LTPA	346,177	12	0.83 (0.75-0.92)	81.8	< 0.001
TPA	7,798	2	1.00 (0.99-1.00)	47.9	0.166
**Adjustments**					
**Smoking adjustment**					
Yes	83,410	7	0.80 (0.66-0.98)	72.2	0.001
No	270,565	7	0.96 (0.93-0.99)	88.2	< 0.001
**Alcohol adjustment**					
Yes	265,393	6	0.83 (0.67-1.02)	74.7	0.001
No	88,582	8	0.96 (0.93-0.99)	86.4	< 0.001
**BMI adjustment**					
Yes	271,046	8	0.88 (0.80-0.96)	75.1	< 0.001
No	82,929	6	0.97 (0.93-1.01)	87.1	< 0.001

OR: odds ratio; CI: confidence interval; LTPA: leisure time physical activity; TPA: total physical activity; BMI: body mass index.

**Figure 6:**
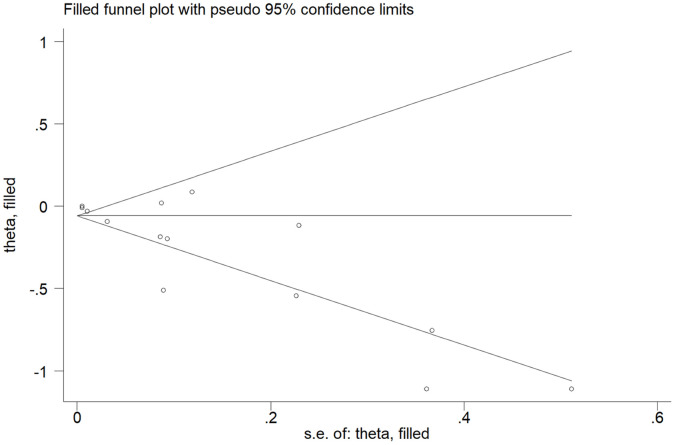
**The funnel plot with trim and fill analysis for studies on the association between PA and CKD risk.**

The Egger’s test showed that there was potential publication bias (*t* = -3.92 and *P* = 0.002). Therefore, trim and fill analysis was conducted for the adjustment of funnel plot asymmetry; no missing studies were added and the adjusted OR was 0.94 (95% CI: 0.91-0.98), which suggested that the pooled OR and 95% CI remained unchanged. The funnel plot of trim and fill analysis was shown in Figure 6.

## DISCUSSION

To our knowledge, this is the first meta-analysis to investigate the dose-response relationship between PA and the risk of CKD. We found that the highest PA level vs. the lowest PA level was associated with reduced odds of CKD. Furthermore, dose-response analysis demonstrated a trend of linear inverse association between PA and the odds of CKD, with a reduction of 2% in CKD risk for every 10 MET h/wk increment of PA.

The current study was consistent with previous studies investigating PA and the risk of several other chronic diseases. Published meta-analysis have showed that higher PA level was associated with reduced risk of hypertension (OR = 0.94, 95% CI = 0.92-0.96) [10], type 2 diabetes (OR = 0.65, 95% CI = 0.59-0.71) [11], metabolic syndrome (OR = 0.74, 95% CI = 0.68–0.80) [12], gestational diabetes mellitus (OR = 0.62, 95% CI = 0.41-0.94) [28], heart failure (OR = 0.70, 95% CI = 0.67-0.73) [13], and Parkinson’s disease (OR = 0.79, 95% CI = 0.68-0.91) [29]. In addition, previous studies also indicated that higher level of PA was associated with decreased risk of mortality [30–33]. Therefore, these findings, as well as the results identified in our study, further suggested that PA has potential beneficial health effects, which might provide relevant population-based evidences for PA preventing chronic diseases including CKD. What’s more, we observed a trend of linear dose-response relationship between PA level and the risk of CKD, which suggested that although current guideline-recommended minimum levels of PA may be sufficient to mitigate health risk, considerably higher levels of PA might be needed to achieve more remarkable reductions in risk of CKD.

Several hypotheses have been developed to explain the potential biological mechanisms linking PA with CKD development. On the one hand, PA may improve vessels endothelial function and slow the atherosclerotic process [34], which may consequently improve renal function. On the other hand, PA also improved insulin sensitivity while habitually low PA could contribute to insulin resistance, which might directly damage renal blood vessels (e.g., angiogenesis, mesangial dilatation, and glomerular high filtration), and adversely affect the kidney by enhancing insulin response signals [35]. Moreover, the beneficial effects of PA on renal health might be partially explained by the reduction in adiposity or in adipocytokines [36].

This meta-analysis had several important strengths. First, this study was a quantitative and comprehensive dose-response meta-analysis to assess the relationship between PA and the risk of CKD. Second, we simultaneously conducted meta-regression and subgroup analysis to explore the potential source of heterogeneity. Third, the “leave-one-out” sensitivity analyses found that no individual specific study significantly affected the pooled OR. Finally, trim and fill analysis did not find any potential missing studies, the pooled OR remained unchanged after adjusting the asymmetry of funnel plot, which indicated that our results were robust and sound.

Our findings should also be interpreted cautiously with regard to some limitations. First, the current meta-analysis was based on observational studies, which make it difficult to establish a causal relationship and rule out the unmeasured confounders. However, we conducted a subgroup analysis based on study design, which suggested the robustness of our findings. In addition, most of the studies included in the current meta-analysis have adjusted several known confounding factors which can reduce the effect of unmeasured confounders. Second, half of the included studies was cross-sectional design, which might introduce recall bias and selection bias. Third, most of the studies measured the PA exposure by self-administrated questionnaires rather than International Physical Activity Questionnaire. Some studies might misclassify the PA levels due to the measurement errors and accuracy inherent in the questionnaires. However, the types of misclassification may be random, and the measurement bias tends to be null. Fourth, only a few studies (7 out of 14) were included in dose-response analysis, the corresponding sample size was therefore relatively small. The finding should be further validated in future investigations.

## CONCLUSIONS

The current meta-analysis suggested that higher level PA is associated with decreased risk of CKD. Future studies comparing the beneficial effects of different PA doses are warranted to determine the optimum dose of PA required for CKD prevention.

## MATERIALS AND METHODS

### Literature search strategy

The study was conducted based on the reporting items of the guidelines for systematic evaluation and meta-analysis (PRISMA). The electronic databases PubMed and Embase were searched up to March 11, 2020 for all publications investigating the association between PA and CKD risk in adults. The following search terms were used: (physical activity OR exercise OR sports OR motor activity OR locomotion fitness OR exercise test OR inactivity OR sedentary activity) AND (chronic kidney disease OR CKD OR kidney disease OR kidney failure OR kidney function OR kidney insufficiency OR kidney dysfunction OR renal disease OR renal failure OR renal function OR renal insufficiency OR renal dysfunction). Furthermore, we also searched the references lists of all relevant reviews and research articles to identify additional eligible studies. All included publications were restricted in English.

### Study selection

Observational studies were included if they met the following criteria: (1) investigated the relationship between PA and the risk of CKD; (2) conducted among adults (age ≥ 18 yrs); (3) estimated effects (relative risk [RR], hazard ratio [HR], or OR) with 95 % CI) were reported or could be calculated. Moreover, for dose–response analysis, a quantitative measure of 3 categories of PA and total number of cases, exposed person-years/participant numbers for each category should be available. If multiple articles reported data from the same population, the ones published in the most recent or with the largest sample size would be included.

### Data extraction and exposure harmonization

Two authors (Y.J.B. and G.F.Z.) independently extracted the following data: the first author’ surname, year of publication, country, study design, definition of CKD, PA level, method and unit of PA assessment, number of cases and person-year/overall participants, HRs/RRs/ORs with 95% CI for each category of PA and covariates adjusted. If more than one type of PA were reported, the LTPA was firstly considered. The disagreements were resolved by a third author (D.S.Z.). The standardized critical appraisal instruments, namely the Newcastle–Ottawa Scale (NOS) [37] and the Joanna Briggs Institute Meta-Analysis of Statistics Assessment and Review Instrument (JBI-MAStARI) [38] were used to assess the quality of cohort study and cross-sectional study, respectively.

For studies [15, 19, 20, 23, 25] set the highest category of PA as reference, we recalculated the risk estimates using the lowest PA category as the reference [39]. For dose-response relationship, the median or mean PA volume (MET h/wk) for each category was assigned to each corresponding HRs/RRs/ORs with 95% CI. When PA was reported by range, the mid-point of the range was used. If the highest category was open-ended, the width of the category was considered to be the same as that of the closet category [40]. When the lowest category was open-ended, the lowest boundary was considered as zero [41]. For the study [23] reported PA only as frequency of min/week, we assumed that the intensity was 4.5 METs, and calculated it into MET h/wk by multiplying the median frequency of the reported category by 4.5 METs [10].

### Statistical analysis

The random effects models were used to assess summary ORs and 95 % CIs for association between the highest versus the lowest level of PA and the risk of CKD. The *χ^2^* and *I^2^* tests were used to evaluate heterogeneity, with *P* < 0.05 and/or *I^2^* > 50% representing significant heterogeneity. We used meta-regression to identify the source of heterogeneity [42]. If no significant covariates were found to be associated with the heterogeneous, the “leave-one-out” sensitivity analysis was used to explore the key studies that have substantial impact on between-study heterogeneity [43]. Subgroup analysis was conducted to investigate the potential modifiable variables of geographic locations, sample size, study design, type of PA, and covariates (smoking, alcohol drinking, and BMI). Begger’s Funnel plots and Egger’s tests were used to evaluate the publication bias with *P* < 0.05 as significant [44, 45].

The generalized least squares regression model, which considered the covariance for each exposure category within each study [46, 47], was used to estimate the dose-response relationship. First, the study specific effect estimates were calculated for per 10 MET h/wk of PA increment, and the pooled effects were synthesised with DerSimonian and Laird random method [48]. In addition, the potential nonlinear relationships were examined using a restrict cubic spline model with 3 knots at 25^th^, 50^th^, and 75^th^ percentiles of the distribution [49]. The non-linear *P* value was calculated by testing the null hypothesis that the second spline coefficient was equal to zero. All statistical analyses were performed by using Stata version 11.0 (StataCorp, College Station, TX, USA).

## Supplementary Material

Supplementary Table 1

Supplementary Tables 2 and 3
