# Supplementary material for: Association of physical activity with chronic kidney disease: a systematic review and dose-response meta-analysis
Source: Aging (Albany NY). 2020 Oct 7;12(19):19221–32. doi: 10.18632/aging.103747 (PMC7732321; doi:10.18632/aging.103747)
Supplement: Supplementary Table 1 [file aging-12-103747-s001..docx]

**Supplementary Table 1. Summary of the characteristics of included studies investigating the association of physical activity (PA) and risk of CKD.**

| Author, year | Country | Study design | CKD definition | Sample size | No. of CKD | Method for PA assessment | Type of PA | PA assessment (dose in MET h/wk) | Reported HR/RR/OR (95% CI) | Covariates adjusted |
| --- | --- | --- | --- | --- | --- | --- | --- | --- | --- | --- |
| Hallan, 2006 [20] | Norway | Cross-sectional | eGFR < 45 mL/min/1.73 m^2^ | 65,193 | 621 | Self-designed questionnaire | LTPA | Physical activity (yes vs no) | 0.60 (0.50-0.71) | Age, sex |
| White, 2011 [23] | Australia | Cohort | eGFR < 60 mL/min/1.73 m^2^ | 10,966 | 958 | Self-designed questionnaire | LTPA | 0 min/week | 1.00 | Age, sex, prevalent diabetes, use of blood pressure lowering medications, systolic blood pressure (mm Hg), serum total cholesterol, prior stroke, prior heart attack, prior angina, current smoking, average alcohol consumption 30 g ethanol per day, BMI |
|  |  |  |  |  |  |  |  | <150 min/week | 1.00 (0.88-1.14) |  |
|  |  |  |  |  |  |  |  | ≥ 150 min/week | 1.02 (0.86-1.21) |  |
| Wakasugi, 2012 [18] | Japan | Cohort | Dipstick urinalysis score of 1+ or greater proteinuria (equivalent to ≥ 30mg/dL) | 4,902 | 110 | Self-designed questionnaire | LTPA | Regular exercise (yes vs. no) | 0.89 (0.57-1.40) | Age, sex, hypertension, diabetes,  hypercholesterolemia |
| Bharakhada, 2012 [17] | United Kingdom | Cross-sectional | The presence of microalbuminuria and/or moderately to severely decreased kidney function (eGFR < 60 mL/min/1.73 m^2^) | 5,656 | 1,071 | International  Physical Activity Questionnaire | LTPA | Low | 1.00 | Age, ethnicity, smoking status, social deprivation, diabetes, cardiovascular disease, systolic blood pressure, relevant medication status, MVPA or sitting time, BMI |
|  |  |  |  |  |  |  |  | Moderate | 0.82 (0.68-0.99) |  |
|  |  |  |  |  |  |  |  | High | 0.82 (0.68-0.98) |  |
| Lee, 2013 [19] | Korea | Cross-sectional | eGFR of 15.0-59.9 mL/min/1.732 m^2^ | 1,239 | 180 | Self-designed questionnaire | LTPA | Regular exercise with strenuous intensity vs. no | 0.47 (0.23-0.97) | Gender, age,  drinking,  smoking, diet with proper amount, exercise, and experience of  having education for DM, hypertension,  hyperlipidemia, IHD, anemia, obesity |
| Chudek, 2014 [25] | Poland | Cross-sectional | eGFR < 60 mL/min/1.73 m^2^ and/or ACR ≥ 30 mg/g | 3,797 | 1,384 | Self-designed questionnaire | LTPA | Regular physical activity in the past (yes vs no) | 1.09 (0.86-1.37) | Gender, age, place of residence, education level, type of work, poverty, smoking status, alcohol consumption, and HF |
| Lin, 2014 [22] | China | Cohort | ICD-9-CM = 580–589 and 250.4 | 559 | 40 | Self-designed questionnaire | LTPA | yes vs. no | 0.33 (0.16-0.66) | Age, sex, BMI, drinking, smoking, dyslipidemia drug, hypertension drug, rice/noodle, meat/livestock, seafood, egg, milk, soybean, vegetables, fruit |
| Jafar, 2015 [21] | China | Cohort | Met at least one of the following criteria: 1) serum creatinine level of more than or equal to 500 μmol/L (5.7 mg/dL), 2) eGFR < 15 ml/min/1.73 m^2^, 3) undergoing hemodialysis or peritoneal dialysis, and 4) had undergone kidney transplant. Criteria 1 to 3 had to be persistent for more than 3 months for qualifying as ESKD. | 59,552 | 10,450 | Self-designed questionnaire | LTPA | Never | 1.00 | Age, sex, interview year, body mass index, dialect, education level, self-reported history of physician diagnosis, hypertension, diabetes, heart disease or stroke, alcohol use, smoking, intake of ginseng, protein intake |
|  |  |  |  |  |  |  |  | ≥ 2 hrs moderate activity only | 0.81 (0.65-1.01) |  |
|  |  |  |  |  |  |  |  | Strenuous aerobic activities | 0.58 (0.37-0.90) |  |
| Hawkins, 2016 [26] | USA | Cohort | eGFR of < 60 mL/min/1.73 m^2^ | 2,435 | 338 | Self-designed questionnaire | LTPA | 0-48 kcal/kg/wk | 1.00 | Age, sex, race, smoking status, baseline Health ABC performance score, study site, use of hypertensive medication, diabetes, CHD, HF, pulse pressure, BMI, HDL, triglycerides, CRP, physical activity, vice versa |
|  |  |  |  |  |  |  |  | 48-93 kcal/kg/wk | 0.97 (0.82-1.13) |  |
|  |  |  |  |  |  |  |  | > 93 kcal/kg/wk | 0.83 (0.70-0.98) |  |
| Michishita, 2016 [15] | Japan | Cross-sectional | eeGFR < 60 ml/min/1.73 m^2^ and/or proteinuria (1+ or greater) | 445 | 39 | Self-designed questionnaire | LTPA | Habitual moderate exercise: ≥ 30 min/once and ≥ 2 times/week (yes vs no) | 0.33 (0.12-0.89) | Age, body mass index, smoking habit, drinking habit, triglyceride, HbA1c, systolic and diastolic blood pressure |
| Alkerwi, 2017 [24] | Luxembourg | Cross-sectional | eGFR < 60 ml/min/1.73 m^2^ (G3-G5), and/or ACR > 30 mg/g Cr (level A2-A3) | 1,359 | 89 | International  Physical Activity Questionnaire | LTPA | 5 MET-hour/week increase | 0.97 (0.95-0.99) | Age, sex, education level, BMI, waist  circumference, HDL-c, triglycerides, serum ALT, hypertension, diabetes |
| Inoue, 2017 (men) [16] | China | Cross-sectional | eGFR < 60 mL/min/1.73 m^2^ | 3,644 | 233 | Self-designed questionnaire | TPA | 10 METs increase | 0.99 (0.98-1.00) | Age, educational attainment,  household income |
| Inoue, 2017 (women) [16] | China | Cross-sectional | eGFR < 60 mL/min/1.73 m^2^ | 4,154 | 399 | Self-designed questionnaire | TPA | 10 METs increase | 1.00 (0.99-1.01) | Age, educational attainment,  household income |
| Guo, 2020 [27] | China | Cohort | eGFR < 60 mL/min/1.73 m^2^ | 190,074 | 10,596 | Self-designed questionnaire | LTPA | Very low-PA | 1.00 | Demographic factors (age, sex and education), baseline eGFR lifestyle factors (physical labour at work, smoking status, alcohol consumption  and vegetable and fruit intake), calendar season and year, BMI, hypertension, diabetes, dyslipidaemia and self-report of a physician diagnosis of CVD and cancer, baseline urinary protein level |
|  |  |  |  |  |  |  |  | Low-PA | 0.93 (0.88-0.98) |  |
|  |  |  |  |  |  |  |  | Moderate-PA | 0.94 (0.89-0.99) |  |
|  |  |  |  |  |  |  |  | High-PA | 0.91 (0.85-0.96) |  |

CKD: chronic kidney disease; PA: physical activity; MET: metabolic equivalent; HR: hazard ratio; RR: relative risk; OR: odds ratio; CI: confidence interval; LTPA: leisure time physical activity; eGFR: estimated glomerular filtration rate; ACR: albumin/creatinine ratio; ICD: international classification of diseases; ESKD: end stage kidney disease; TPA: total physical activity; MVPA: moderate to vigorous intensity physical activity; BMI: body mass index; DM: diabetes mellitus; IHD: ischemic heart disease; HF: heart failure; CHD: coronary heart disease; HDL: High density lipoprotein: CRP: c-reaction protein; CVD: cardiovascular disease.
