# Supplementary material for: Association of physical activity with chronic kidney disease: a systematic review and dose-response meta-analysis
Source: Aging (Albany NY). 2020 Oct 7;12(19):19221–32. doi: 10.18632/aging.103747 (PMC7732321; doi:10.18632/aging.103747)
Supplement: Supplementary Tables 2 and 3 [file aging-12-103747-s002..pdf]

SUPPLEMENTARY TABLES

Supplementary Table 2. Quality assessment of cohort studies.

Author, year	NOS								Total
	Selection				Comparability	Outcome			
	Representativeness of the exposed cohort	Selection of the non-exposed cohort	Ascertainment of exposures	Demonstration that outcome of interest was not present at start of study	Comparability of cohorts on the basis of the design or analysis controlled for confounders	Assessment of outcome	Was follow up long enough for outcomes to occur	Adequacy of follow up of cohort	
White, 2011 [1]	1	1	1	1	1	1	1	1	8
Wakasugi, 2012 [2]	0	1	0	1	1	1	1	1	6
Lin, 2014 [3]	1	1	1	1	1	1	1	0	7
Jafar, 2015 [4]	1	1	1	1	1	1	1	1	8
Hawkins, 2016 [5]	0	1	1	0	1	1	1	1	6
Guo, 2020 [6]	1	1	1	1	1	1	1	1	8

NOS: Newcastle–Ottawa Scale.

Supplementary Table 3. Quality assessment of cross-sectional studies.

Author, year	Were the criteria for inclusion in the sample clearly defined	Were the study subjects and the setting described in detail	Was the exposure measured in a valid and reliable way	Were objective, standard criteria used for measurement of the condition	Were confounding factors identified	Were strategies to deal with confounding factors stated	Were the outcomes measured in a valid and reliable way	Was appropriate statistical analysis used
Hallan, 2006 [7]	Yes	Yes	No	No	Yes	Yes	Yes	Yes
Bharakhada, 2012 [8]	Yes	Yes	Yes	Yes	Yes	Yes	Yes	Yes
Lee, 2013 [9]	Yes	Yes	Yes	Yes	Yes	Yes	Yes	Yes
Chudek, 2014 [10]	Yes	Yes	No	No	Yes	Yes	Yes	Yes
Michishita, 2016 [11]	Yes	Yes	Yes	Yes	Yes	Yes	Yes	Yes
Alkerwi, 2017 [12]	Yes	Yes	Yes	Yes	Yes	Yes	Yes	Yes
Inoue, 2017 [13]	Yes	Yes	No	No	Yes	Yes	Yes	Yes

JBI-MAStARI: Joanna Briggs Institute Meta-Analysis of Statistics Assessment and Review Instrument.
